# MicroRNA-195-5p is a potential diagnostic and therapeutic target for breast cancer

**DOI:** 10.3892/or.2014.2971

**Published:** 2014-01-08

**Authors:** QIFENG LUO, CHUANKUI WEI, XIAOYU LI, JIA LI, LEI CHEN, YIXIANG HUANG, HONGMING SONG, DENGFENG LI, LIN FANG

**Affiliations:** 1 Department of General Surgery, Shanghai Tenth People’s Hospital, Tongji University School of Medicine, Shanghai 200072, P.R. China; 2 Department of Microbiology and Genetic Institute, Paris-Sud 11 University, Paris, France

**Keywords:** microRNA, miR-195-5p, breast cancer

## Abstract

MicroRNAs (miRNAs) are a class of highly conserved, small endogenous single-strand non-coding RNAs. They are aberrantly expressed in the circulation and tissue of patients with cancer. Therefore, it has been suggested that they may act as key regulators of carcinogenesis. The aim of the present study was to examine the expression level of miR-195-5p in human breast cancer and its potential role in carcinogenesis. The expression level of miR-195-5p was measured in 40 breast cancer specimens and adjacent normal breast tissues by quantitative polymerase chain reaction (qPCR). Next, to explore the potential function of miR-195-5p, we used MDA-MB-231 human breast cancer cells and carried out MTT, colony formation, Transwell chamber migration and cell cycle assays. The dual-luciferase reporter assay was also performed to determine putative targets of miR-195-5p, which were validated using qPCR and western blot assays. We found that miR-195-5p expression was significantly decreased in the 40 breast cancer specimens when compared with that in the adjacent normal breast tissues (P<0.05). Overexpression of miR-195-5p inhibited cell proliferation, reduced cell colony formation, suppressed cell migration and caused an accumulation of cells in the G1 phase of the cell cycle. In the 3′-untranslated region (3′-UTR) of cyclin E1 (CCNE1), we found two putative target sites which may bind miR-195-5p, suggesting that CCNE1 is a direct target of miR-195-5p. Furthermore, through qPCR and western blot assays we showed that overexpression of miR-195-5p reduced CCNE1 mRNA and protein levels, respectively. Our study suggests that miR-195-5p may act as a tumor suppressor in breast cancer. Therefore, targeting of this miRNA may provide a novel strategy for the diagnosis and treatment of patients with this lethal disease.

## Introduction

Breast cancer is the most common malignant disease and accounts for 14% of female cancer-related deaths worldwide each year. Indeed, although diagnostic and therapeutic methods have greatly improved over the last decade, this cancer remains the leading cause of cancer-related death among females (1). Therefore, it is essential to develop more effective methods for its early diagnosis and treatment.

MicroRNAs (miRNAs) are a small class of non-coding RNAs which regulate gene expression and may play pivotal roles in the physiological and pathological processes in a variety of eukaryotic organisms (2). miRNAs achieve their effects through base-pairing with the 3′-untranslated region (3′-UTRs) of target mRNAs, which can lead to the translational repression or mRNA degradation (3,4). It is known that miRNAs are involved in tumor cell proliferation, migration, invasiveness and metastasis (5). Moreover, aberrant miRNA expression has been frequently observed in various types of human tumors. These reports suggest that miRNAs may function as either tumor-suppressor genes or oncogenes (6). In human breast cancer, several miRNAs such as let-7 (7), miR-155 (8) and miR-200 have been shown to be dysregulated (9). A recent study showed that miR-195 is significantly downregulated in breast cancer (10). However, the role that miR-195-5p plays in the carcinogenesis of breast cancer is still largely unknown.

Cyclin E1 (CCNE1) belongs to the cyclin family which, through association with cyclin-dependent kinase 2, controls the progression of the cell cycle by driving cells from the G1 to the S phase (11). Previous studies have shown that CCNE1 is aberrantly expressed and may function as an oncogene in many types of human cancers (12,13). Furthermore, significant evidence indicates that breast cancer patients with higher levels of CCNE1 show a higher mortality rate when compared with those bearing low CCNE1 levels (14–16). Thus, CCNE1 has attracted increasing research interest.

In the present study, we initially demonstrated that the expression level of miR-195-5p in breast cancer specimens was significantly lower than that in adjacent normal tissues. Cell functional studies further showed that overexpression of miR-195-5p inhibited proliferation and colony formation ability, suppressed cell migration and caused G1 phase arrest by targeting CCNE1 in MDA-MB-231 breast cancer cells. Therefore, our study suggests that miR-195-5p may act as a tumor suppressor, and thus may be considered a potential therapeutic and diagnostic target of breast cancer.

## Materials and methods

### Specimens

A total of 40 breast cancer specimens and matched adjacent normal breast tissues were surgically obtained from patients at the Department of General Surgery of the Shanghai Tenth People’s Hospital. Collection of the patient specimens was approved by the Institutional Ethics Committee of Tongji University. These samples were snap frozen in liquid nitrogen. All samples were confirmed as invasive, ductal breast cancer by pathologists. None of the patients received radiotherapy or chemotherapy prior to surgery.

### Cell culture and transfection

Human MDA-MB-231 breast cancer and HEK293T cells were purchased from the Chinese Science Institute (Shanghai, China). The cells were cultured in Dulbecco’s modified Eagle’s medium (DMEM) supplemented with 10% fetal bovine serum (FBS) (both from Gibco, USA), penicillin (100 U/ml) and streptomycin (100 μg/ml) (Enpromise, Hangzhou, China) at 37°C with 5% CO_2_ in saturated humidity. Cells in the logarithmic growth phase (~80% confluence) were selected for the experiments. Those with over 95% viability as shown by trypan blue staining were qualified for further experiments.

miR-195-5p mimics and non-specific negative control (NC) oligos were purchased from GenePharma (Shanghai, China). The sequence of the miR-195-5p mimic was 5′-UAGCA GCACAGAAAUAUUGGC-3′ and the sequence of the NC mimic was 5′-UCACAACCUCCUAGAAAGAGUAGA-3′. For transfection, MDA-MB-231 cells (2×10^5^) were added into each well of a 6-well plate and cultured with serum- and antiobiotic-free DMEM. When the cell density achieved 30–40% confluence, Lipofectamine transfection reagent (Invitrogen, USA) was used to introduce the mimics according to the manufacturer’s instructions. The ratio of mimics to Lipofectamine was 1 μg to 3 μl.

### miRNA isolation and quantitative polymerase chain reaction (qPCR)

miRNAs were extracted from the tissues using the miRcute microRNA isolation kit (Tiangen, Beijing, China) according to the manufacturer’s instructions. The expression level of miR-195-5p was detected by the One-Step qRT-PCR method (EzOmics SYBR qPCR kit). The miR-195-5p primer, U6 primer and EzOmics SYBR qPCR kit were purchased from Biomics Biotechnologies Inc. (Jiangsu, China). The U6 primer used as an internal control was: 5′-GTCCTATCCAGTGCAGGGTCC GAGGTGCACTGGATACGACAAAATATGGAAC-3′ (stem-loop primer) 5′-TGCGGGTGCTCGCTTCGCAGC-3′ (sense) and 5′-CCAGTGCAGGGTCCGAGGT-3′ (antisense). Briefly, for amplification of miR-195-5p, 100 ng RNA was used in a 25-μl reaction system containing 12.5 μl 2X Master Mix, 0.5 μl 50X SYBR-Green, 0.5 μl reverse transcription primer (10 μM), 0.5 μl sense and 0.5 μl antisense primers (10 μM). One Step PCR parameters for miRNA quantification were as follows: 37°C for 60 min for reverse transcription, 10 min at 95°C, and then 40 cycles of 20 sec at 95°C, 30 sec at 62°C and 30 sec at 72°C. Each sample was tested in triplicate.

For RNA analysis, total RNA was isolated from the cultured cells using TRIzol reagent (Invitrogen, USA) according to the manufacturer’s instructions. For CCNE1 mRNA detection, reverse transcription was performed using the PrimeScript RT-PCR kit (Takara, Shiga, Japan). Real-time PCR was performed using a 7900HT Fast RT-PCR instrument (Applied Biosystems, Singapore) using SYBR-Green. GAPDH mRNA levels were used for normalization. The primer sequences were as follows: CCNE1, 5′-GTGTGGGAGCCAGCCTTG-3′ (sense) and 5′-ATCATCTTCTTTGTCAGGTGTGG-3′ (antisense); GAPDH, 5′-AAGGTCGGAGTCAACGGATT-3′ (sense) and 5′-CTGGAAGATGGTGATGGGATT-3′ (antisense). The PCR parameters for relative quantification were as follows: 5 min at 94°C, followed by 30 cycles of 30 sec at 94°C, 45 sec at 57°C and 45 sec at 72°C. Each sample was tested in triplicate, and the fold-change of mRNA expression was calculated using the 2^−ΔΔCt^ method (17).

### Cell proliferation [3-(4,5-dimethylthiazol-2-yl)-2,5-diphenyltetrazolium bromide (MTT)] assay

Cells were plated at 3,000/well in 96-well plates (BD Biosciences, USA) and incubated at 37°C. When the cells reached 30–40% confluence, they were transfected with either 50 or 100 nM miR-195-5p mimics or NC mimics. One group of cells was treated with lipofectamine alone as a mock control. We then assessed cell proliferation at 24, 48, 72 and 96 h post-transfection using the MTT assay. Briefly, 20 μl (5 mg/ml) MTT (Sigma, USA) solution was added to each well. After a 4-h incubation at 37°C, the supernatant was removed and 150 μl DMSO was added. After 10 min of agitation (100 rpm), the absorbance at 490 nm of each sample was measured by a microplate spectrophotometer. Each experiment was performed in triplicate and included 6 replicates.

### Colony formation assay

Three hundred cells of each group (miR-195-5p, mock and NC) were plated in a 6-well plate in complete medium. After incubation at 37°C with 5% CO_2_ for 7–10 days, or when the colonies were visible by viewing with the eye, the culture was terminated. Complete medium was removed, and the plates were washed twice in phosphate-buffered saline (PBS). The colonies were fixed in 95% ethanol for 10 min, dried and stained with 0.1% crystal violet solution for 10 min. Next, each plate was washed three times with water, and the number of colonies was counted only if the well contained >50 cells. The experiment was performed three times.

### Transwell migration assay

The Transwell migration assay was performed to evaluate cell migration ability. First, the filters (Corning, Lowell, MA, USA) were washed with serum-free DMEM, and placed into a 24-well plate. The lower chambers contained DMEM with 10% FBS. For the upper chambers, 3×10^4^ cells were resuspended in 200 μl DMEM with 0.1% BSA. Plates were then incubated at 37°C in 5% CO_2_. After 20 h, the cells that migrated through the membranes were fixed with methanol and stained with crystal violet. Images of six randomly selected fields-of-view were captured, and the cells were counted.

### Cell cycle assay

miR-195-5p (100 nM), mock and NC cells were harvested at 48 h after transfection, centrifuged at 1,200 rpm for 10 min and washed three times with cold PBS. Ice-cold 70% ethanol was subsequently added dropwise, and the cells were fixed at 4°C overnight. After a 30-min digestion in RNase (0.1 g/l), a total of 250 μl (0.05 g/l) propidium iodide (PI) staining solution was added to each sample which was then incubated for 30 min at room temperature (RT) in the dark. The cell cycle was then analyzed by a flow cytometer (FACSCanto™ II; BD Biosciences).

### Dual-luciferase reporter assay

293T cells were seeded in 12-well plates (BD, USA) in complete medium and incubated at 37°C with 5% CO_2_. CCNE1 3′-UTR were cloned into the psiCHECK-2 vector, and co-transfected with miR-195-5p mimics (100 nM) or NC mimics when cells reached 80–90% confluence. Thirty-six hours after transfection, luciferase activity was measured by the dual-luciferase reporter assay kit (Promega, USA). Briefly, the cells were washed twice with PBS then lysed by incubation at RT for 15 min with passive lysis buffer (PLB). The supernatants were collected, and 20 μl of the aliquots was added to 96-well plates. The firefly luciferase (FL) reporter was measured immediately after adding Luciferase Assay Reagent II (LAR II). Next, 100 μl of Stop & Glo^®^ reagent was added to each well to initiate the *Renilla* luciferase (RL). The psiCHECK-2 vector that provides constitutive expression of FL was co-transfected as an internal control. All experiments were performed three times.

### Western blot analysis

Cell protein was extracted using RIPA lysis buffer. The supernatant was quantified by bicinchoninic acid assay (Pierce, USA). Next, 25 μg of protein samples was denatured with 5X sodium dodecyl sulfate (SDS) loading buffer at 95°C for 5 min. Subsequently, whole protein samples were separated by 10% SDS-polyacrylamide gel electrophoresis (SDS-PAGE) and transferred onto 0.45-μm nitrocellulose membranes (Beyotime). Following 1 h of blocking in 5% fat-free milk, the membranes were incubated with the CCNE1 antibody (1:1,000) and the β-actin antibody (1:1,000) (both from Epitomics, USA) overnight at 4°C. Blots were then washed and incubated for 1 h with secondary antibodies. After washing with PBST, immunoreactive protein bands were detected using the Odyssey scanning system (LI-COR, Lincoln, NE, USA).

### Statistical analysis

Data from at least three separate experiments are presented as the means ± standard error of the mean (SEM). The two-tailed t-test was used to draw a comparison between groups. Differences were considered significant for P-values <0.05.

## Results

### miR-195-5p expression is decreased in breast cancer specimens

**Figure 1:**
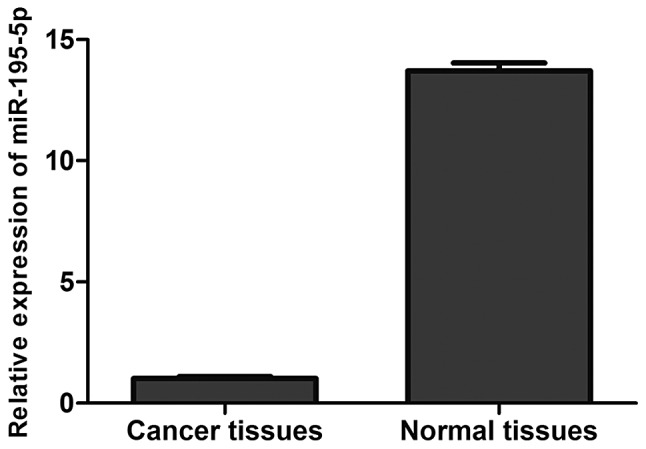
The expression level of miR-195-5p is significantly decreased in the breast cancer tissues. miRNA was extracted from breast cancer and adjacent normal tissues. The expression of miR-195-5p was analyzed by qRT-PCR. The graph represents the 2^−ΔΔCt^ values ± SEM; ^*^P<0.05. SEM, standard error of the mean.

We measured the mRNA expression levels of miR-195-5p in breast cancer specimens and the adjacent normal tissues by real-time PCR. As shown in Fig. 1, compared with the adjacent normal tissues, miR-195-5p expression was significantly decreased in the breast cancer specimens (P<0.05).

### Overexpression of miR-195-5p in MDA-MB-231 cells inhibits cell proliferation and colony formation ability

**Figure 2:**
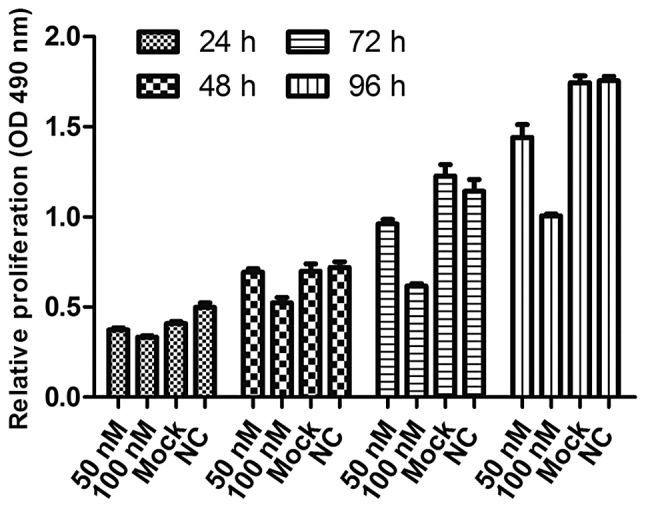
Overexpression of miR-195-5p inhibits cell proliferation. The proliferation of MDA-MB-231 cells transfected with miR-497 (50 and 100 nM) was examined at various time points and compared with that of the mock and NC groups. The graph represents OD 490 ± SEM nm; ^*^P<0.05. NC, negative control; SEM, standard error of the mean.

**Figure 3:**
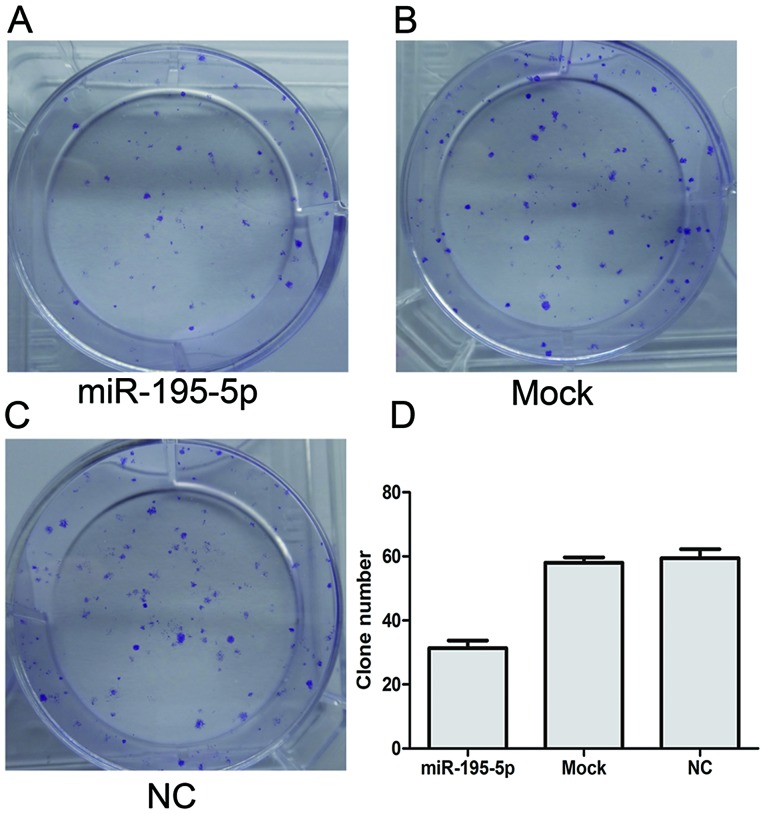
Overexpression of miR-195-5p inhibits cell colony formation ability. (A–C) Representative images of crystal violet-stained colonies in the MDA-MB-231 cells. (A) The miR-195-5p group exhibited fewer colonies than the (B) mock or (C) NC group. (D) Graphical representation of the clone number for each group; ^*^P<0.05. NC, negative control.

The viability of cells transfected with either 50 or 100 nM miR-195-5p mimics was measured and compared with the mock and NC transfected cells at 24, 48, 72 and 96 h post-transfection. We found that the viability of both miR-195-5p mimic groups was consistently significantly lower than the mock and NC groups in a time- and dose-dependent manner (Fig. 2). Thus, 100 nM was used in the following experiments. As shown in Fig. 3, the 100 nM miR-195-5p group exhibited fewer colonies than the mock and NC groups as determined by the colony formation assay. These results suggest that transient overexpression of miR-195-5p suppresses the proliferation and colony formation ability of MDA-MB-231 cells.

### Overexpression of miR-195-5p in MDA-MB-231 cells inhibits cell migratory ability

**Figure 4:**
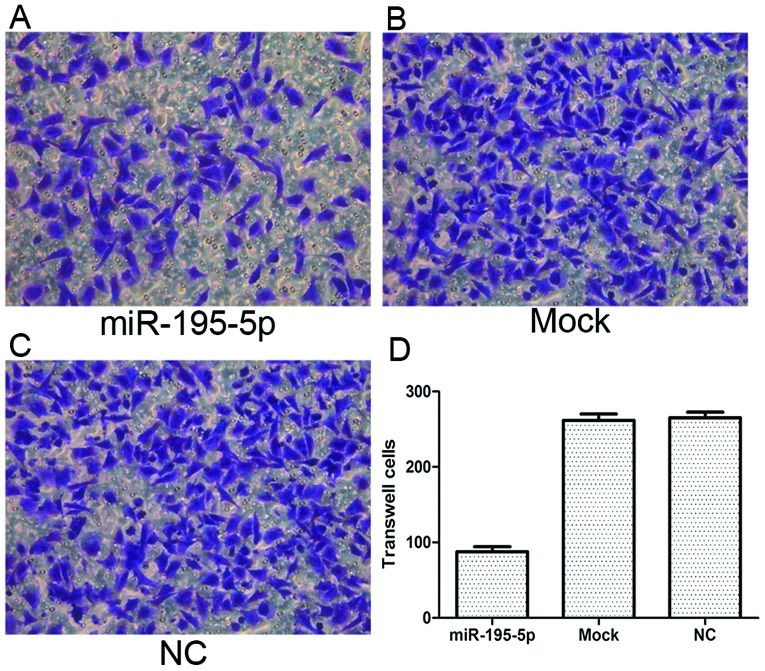
Overexpression of miR-195-5p inhibits cell migratory ability. (A–C) Representative images of crystal violet-stained MDA-MB-231 migratory cells. Images were captured using an inverted microscope with ×200 magnification. The number of migrating cells in the (A) miR-195-5p group was significantly less than that in either the (B) mock or (C) NC group. (D) Quantification of the number of crystal violet-stained cells. Data represent means ± SEM; ^*^P<0.05. NC, negative control; SEM, standard error of the mean.

The cell migratory ability of the MDA-MB-231 cells with and without transfection of miR-195-9p mimics was detected by Transwell migration assay. Our results showed that 20 h after transfection, the number of migrating cells in the miR-195-5p group was significantly less than that in either the mock or NC groups (P<0.05). These data suggest that the migratory ability of MDA-MB-231 cells may be inhibited by miR-195-5p (Fig. 4).

### Overexpression of miR-195-5p initiates G1 phase arrest of MDA-MB-231 cells

**Figure 5:**
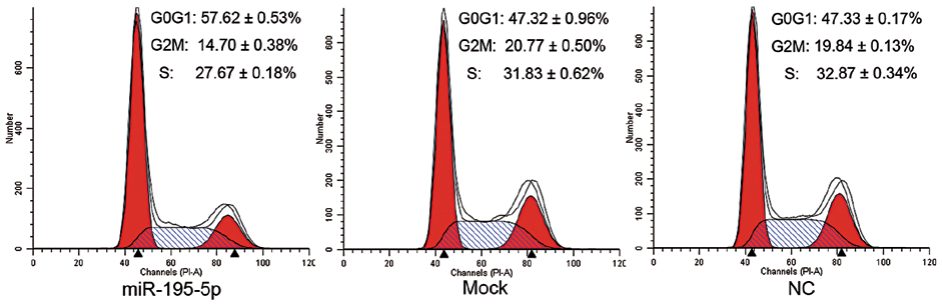
Overexpression of miR-195-5p disrupts the cell cycle distribution. Cell cycle distribution of MDA-MB-231 cells transfected with miR-195-5p mimics, NC or mock, was analyzed by flow cytometry. The respective proportions of G1, S and G2 phase cells are shown, ^*^P<0.05. NC, negative control.

The cell cycle distribution of the MDA-MB-231 cells with and without transfection of miR-195-5p mimics was analyzed by flow cytometry. As shown in Fig. 5, the percentage of cells remaining in the G1 phase in the miR-195-5p overexpression group (57.62±0.53%) was significantly greater than that of the mock (47.32±0.96%) and NC groups (47.33±0.17%, P<0.05); while the proportion of G2 and S phase cells decreased in the miR-195-5p group compared with those of the mock and NC groups (P<0.05). These results indicate that the overexpression of miR-195-5p prevents cells from entering the S phase through initiation of G1 phase arrest in MDA-MB-231 cells.

### miR-195-5p regulates CCNE1 expression by targeting its mRNA in MDA-MB-231 cells

**Figure 6:**
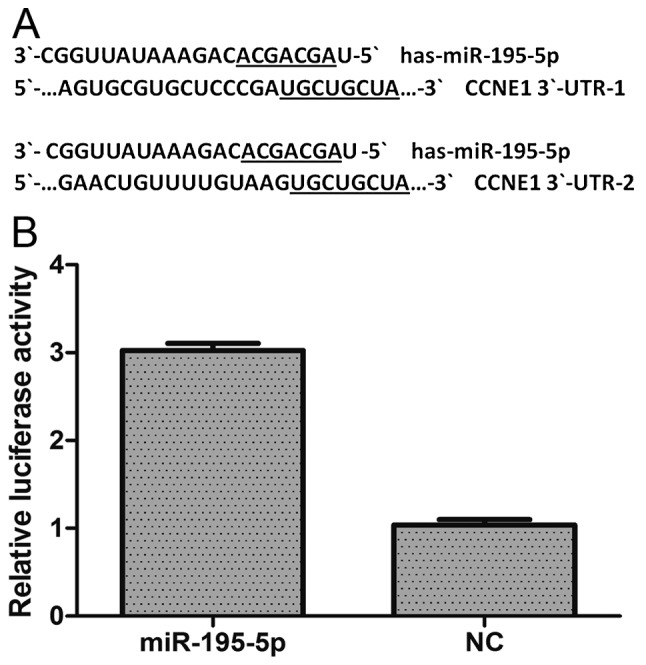
CCNE1 is a direct target of miR-195-5p. (A) The binding sites for miR-195-5p in the 3′-UTR of CCNE1 mRNA. (B) The relative luciferase activity (FL/RL) was measured in HEK293T cells after co-transfection of the CCNE1 luciferase construct with either miR-195 or NC; ^*^P<0.05. CCNE1, cyclin E1; 3′-UTR, 3′-untranslated region; NC, negative control.

**Figure 7:**
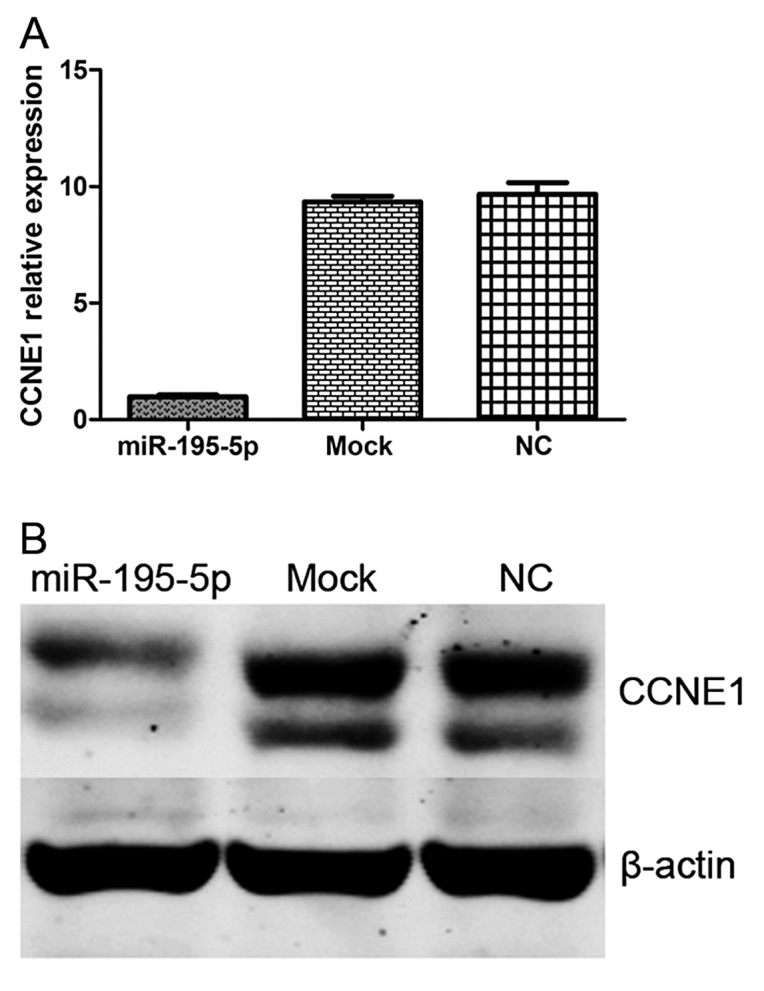
Overexpression of miR-195-5p inhibits CNNE1 expression in MDA-MB-231 cells. (A) qRT-PCR and (B) western blot analysis of CCNE1 mRNA and protein levels, respectively, in MDA-MD-231 cells transfected with miR-195-5p, mock or NC. CCNE1, cyclin E1; NC, negative control.

To validate the possibility that miR-195-5p may target CCNE1, we initially searched for putative targets within its mRNA sequence using three bioinformatic algorithms: miRanda, TargetScan and miRBase. We found two potential binding sites for miR-195-5p which were located 247–254 and 485–492 bp downstream from the 5′ end of the CCNE1 3′-UTR (Fig. 6A). Next we constructed a psiCHECK-2/CCNE1 3′-UTR vector, which contained the *Renilla* luciferase (RL) gene and the 3′-UTR region of CCNE1. This construct was transfected into 293T cells together with either miR-195-5p or NC mimics, and the luciferase activity was analyzed. The ratio of FL/RL was calculated, and showed that the miR-195-5p group had an ~3-fold higher activity than that of the NC group (P<0.05) (Fig. 6B). These results suggest that miR-195-5p directly interacts with the CCNE1 3′-UTR in the psiCHECK-2 reporter plasmid and leads to the degradation of RL mRNA. Finally, we performed qPCR and western blot analysis of CCNE1 expression in MDA-MB-231 cells with and without transfection of miR-195-5p mimics, or controls. We demonstrated that overexpression of miR-195-5p significantly decreased CCNE1 expression at both the mRNA and protein levels (Fig. 7). These data further indicate that CCNE1 is a target of miR-195-5p.

## Discussion

In addition to surgery and traditional chemotherapeutic drugs, several molecularly targeted drugs have been developed for the treatment of breast cancer. The drugs that have been assessed for the treatment and prevention of breast cancer, such as raloxifene, letrozole and exemestane, in preclinical and clinical studies (18), have resulted in a decline in the incidence rate of breast cancer. Over the last decades, miRNA research has become a ‘hot spot’ for research. Recent advances suggest that dysregulation of miRNAs is a common event in human cancers (19–22) and that they may thus act as key regulators of carcinogenesis. Based on these findings, it has been proposed that more effective targets or targeted drugs for diagnosing and treating breast cancer may involve miRNAs.

In the present study, we examined the expression of miR-195-5p in human breast cancer and its potential role in carcinogenesis. First, through qPCR, we found that the expression level of miR-195-5p in breast cancer specimens was significantly lower than that in adjacent normal tissues. This suggests that the expression of miR-195-5p is associated with the development of breast cancer, and that it may function as a tumor suppressor. Indeed, the expression of miRNA-195, which is closely related to miRNA-195-5p, has been previously reported to be decreased in human breast cancer. Meanwhile, upregulation of miR-195 expression has been shown to suppress cell proliferation and invasion by targeting the Raf-1 and Ccnd1 genes in both ZR-75-30 and MCF7 human breast cancer cells (10). miRNA-195 includes miRNA-195-5p and miRNA-195-3p, and together they belong to the miRNA-15 family.

In the present study, we transfected miR-195-5p mimics into MDA-MB-231 cells to generate its overexpression. This exogenous overexpression of miR-195-5p significantly inhibited proliferation and colony formation ability of MDA-MB-231 cells as measured by MTT and colony formation assays, respectively. Moreover, cell migration ability was also significantly reduced by overexpression of miR-195-5p in the MDA-MB-231 cells. Furthermore, by flow cytometry we found that overexpression of miR-195-5p prevented cells from entering the S phase and instead caused an accumulation of cells in the G1 phase.

To ascertain why miR-195-5p exhibited these effects on the cell function, we investigated putative targets of miR-195-5p and identified CCNE1, which drives cells from the G1 to the S phase. Based on three databases, we found that the CCNE1 3′-UTR contains two miR-195-5p matching sites. Notably, the interaction between miR-195-5p and CCNE1 mRNA has not been previously reported. To test whether CCNE1 was a real target of miR-195-5p, we constructed a psiCHECK-2 plasmid containing the 3′-UTR of CCNE1 (psiCHECK-2/CCNE1 3′-UTR). Through dual-luciferase assays, we confirmed that CCNE1 was a direct target of miR-195-5p. Additionally, we found that the mRNA and protein levels of CCNE1 were significantly reduced in miR-195-5p-overexpressing cells when compared with those transfected with either mock or NC, thus further indicating that CCNE1 is a direct target of miR-195-5p.

In summary, overexpression of miR-195-5p inhibited the proliferation and colony formation ability, suppressed migration and caused G1 phase arrest by targeting CCNE1 in MDA-MB-231 breast cancer cells. All of the data suggest that miR-195-5p is a tumor suppressor that may inhibit carcinogenesis in human breast cancer. Therefore, miR-195-5p may be a potential diagnostic and therapeutic target for breast cancer.
