# [Expression of Concern] MicroRNA-195-5p is a potential diagnostic and therapeutic target for breast cancer

**DOI:** 10.3892/or.2026.9200

**Published:** 2026-09-23

**Authors:** Qifeng Luo, Chuankui Wei, Xiaoyu Li, Jia Li, Lei Chen, Yixiang Huang, Hongming Song, Dengfeng Li, Lin Fang

Oncol Rep 31: 1096–1102, 2014; DOI: 10.3892/or.2014.2971

Following the publication of the above article, a concerned author drew to the authors' attention that, regarding the cell migration assay experiments shown in Fig. 4 on p. 1099, Fig. 4B and C, showing the results of the ‘NC’ and ‘Mock’ experiments, contained an overlapping section of data, suggesting that these data panels were derived from the same original source where different experiments were intended to have been portrayed.

The authors have been contacted by the Editorial Office to offer an explanation for this apparent anomaly in the presentation of the data in this paper, and we are awaiting their response. Owing to the fact that the Editorial Office has been made aware of this potential issue concerning the scientific integrity of this paper, we are issuing an Expression of Concern to notify readers of this potential problem while the Editorial Office continues to investigate this matter further.

